# Hypovolemic Shock Revealing a Gastrointestinal Stromal Tumor

**DOI:** 10.7759/cureus.37315

**Published:** 2023-04-08

**Authors:** Fatima Zahra Belabbes, Safa Ibork, Kenza Oqbani, Ahmed Bensaad

**Affiliations:** 1 Gastroenterology and Hepatology, Faculty of Medicine, Mohammed VI University of Health Sciences (UM6SS) Cheikh Khalifa International University Hospital, Casablanca, MAR; 2 Gastroenterology and Hepatology, Faculty of Medicine, Mohammed VI University of Sciences and Health (UM6SS) Cheikh Khalifa International University Hospital, Casablanca, MAR; 3 Pathology, Faculty of Medicine, Mohammed VI University of Sciences and Health (UM6SS) Cheikh Khalifa International University Hospital, Casablanca, MAR; 4 General Surgery, Mohammed VI University of Sciences and Health (UM6SS) Cheikh Khalifa International University Hospital, Casablanca, MAR

**Keywords:** surgey, endoscopy, stromal tumor, gastrointestinal, shock

## Abstract

Gastrointestinal stromal tumors (GISTs) are rare neoplasms that originate in the gastrointestinal tract. Due to the nonspecific symptoms, they are often underdiagnosed. Patients typically present with abdominal pain, weight loss, asthenia, or a sensation of a "ball in the stomach." Hypovolemic shock is a rare mode of presentation. The biopsy is often inconclusive, and immunohistochemistry plays a crucial role in diagnosis. Surgery is the treatment of choice for stromal tumors with hemorrhage.

Here, we present two cases of patients admitted in critical condition with hypovolemic shock. Laboratory results revealed profound anemia. Upper gastrointestinal exploration demonstrated a tumor in both cases, with normal biopsy findings in one case. However, after partial gastrectomy, pathology results revealed GIST with an immunohistochemistry profile in favor. The mode of presentation in our cases is notable, as hypovolemic shock without apparent external bleeding is an unusual presentation. Therefore, physicians should consider GIST a possible diagnosis when presented with a patient in hypovolemic shock, even without externalized bleeding.

## Introduction

Gastrointestinal stromal tumors (GISTs) are mesenchymal tumors of the gastrointestinal tract that arise from a proliferation of Cajal cells. Although rare, they represent the most common sarcoma of the digestive tract [1]. They account for less than 1% of all gastrointestinal tumors and have an estimated incidence of 10-15 cases per million per year [2]. GISTs are primarily located in the stomach (antral, fundic, rarely cardial), intestine, and less frequently in the rectum, colon, esophagus, and mesentery. Despite their prevalence, GISTs are often underdiagnosed due to the nonspecific symptoms they present with. Digestive bleeding is the most common mode of presentation. Immunohistochemistry is an essential diagnostic tool for GIST, with *CD117/KIT*+ (95%) and *DOG-1*+ (95%) phenotypes being the most frequently observed [3]. However, preoperative biopsies are not always conclusive, and symptoms are often nonspecific, leading to underdiagnosis [4].

In conclusion, GISTs are an often underdiagnosed entity with a nonspecific presentation. However, as illustrated in our cases, GISTs should also be considered in patients with hypovolemic shock without any apparent external bleeding [5]. Therefore, clinicians should maintain a high index of suspicion for GISTs and be aware of their varied presentations to ensure early diagnosis and appropriate management. In this report, we present two cases of patients with subcardial and fundic GISTs, respectively, who presented with hypovolemic shock without any visible gastrointestinal bleeding.

## Case presentation

Case 1

A 73-year-old male with no significant medical history and no history of anticoagulant or antiplatelet use was admitted to the hospital for profound asthenia and pallor, with sudden onset of shortness of breath, and profuse sweating, despite having no melena, hematochezia, or hematemesis. He was in hypovolemic shock: arterial pressure at 60/30 mmHg, cardiac frequency at 130 beats per minute (bpm), and polypnea. The patient had a normal abdominal examination and the rest of the clinical examination was unremarkable. Laboratory analysis revealed severe anemia with hemoglobin levels at 5.3 g/dL. The patient was stabilized with transfusions and hemodynamic support.

**Figure 1:**
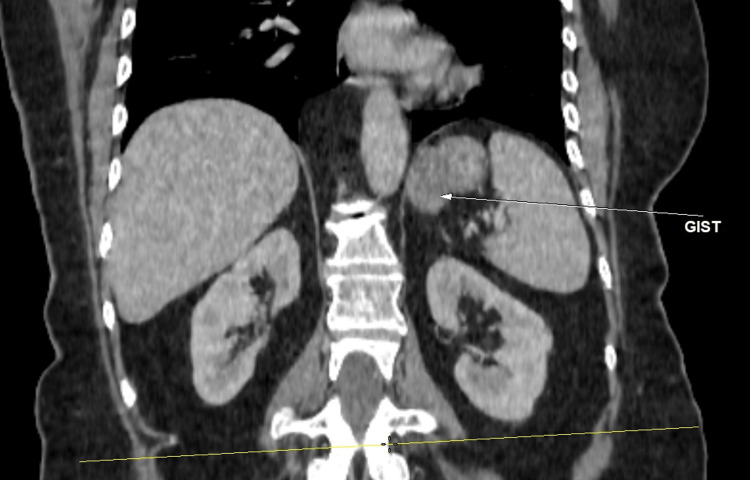
Abdominal CT in the coronal section The image shows a 5 cm solid, exophytic, ovoid mass in the gastric region with no evidence of invasion into adjacent tissues and irregular borders, which is indicative of GIST (arrow).

An abdominal computed tomography (CT) was performed, which revealed an extraluminal tumor mass of the stomach, measuring 50x20 mm with endoluminal development extending over 5 cm, without any evidence of adenopathy or secondary hepatic or pulmonary lesions (Figure 1).

**Figure 2:**
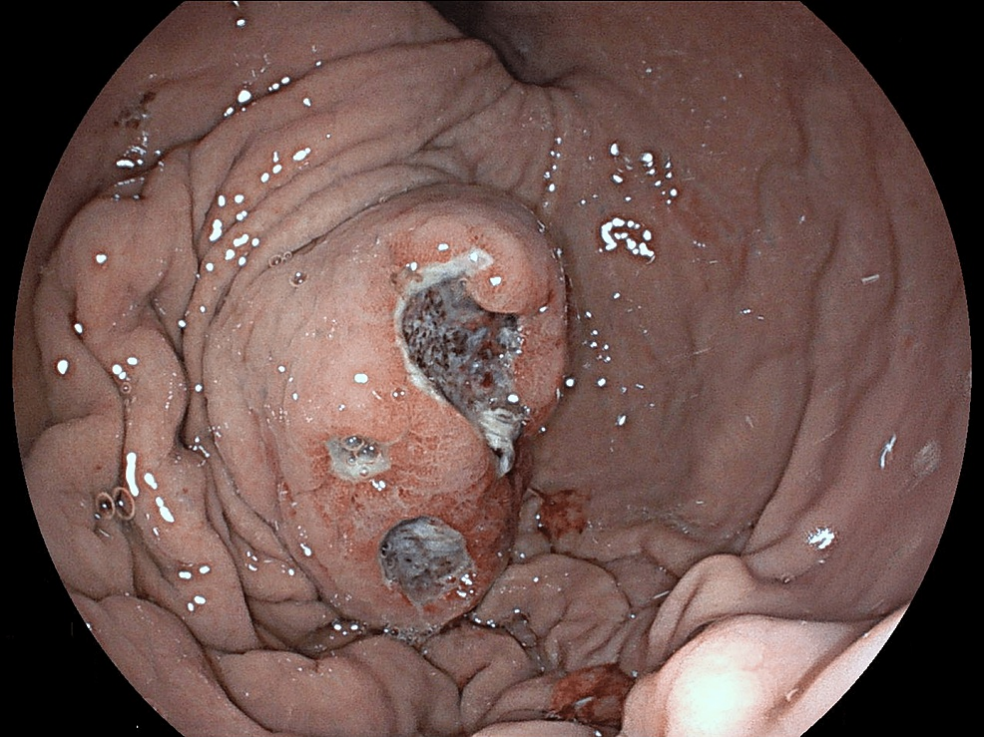
Upper gastrointestinal endoscopy image Upper gastrointestinal endoscopy revealed a 5 cm long formation with extrinsic development, ulcerated at its center.

Upper gastrointestinal endoscopy revealed a 5 cm long polypoid formation with extrinsic development, ulcerated at its center, and fragile at biopsy (Figure 2). Lower gastrointestinal exploration found only melena.

**Figure 3:**
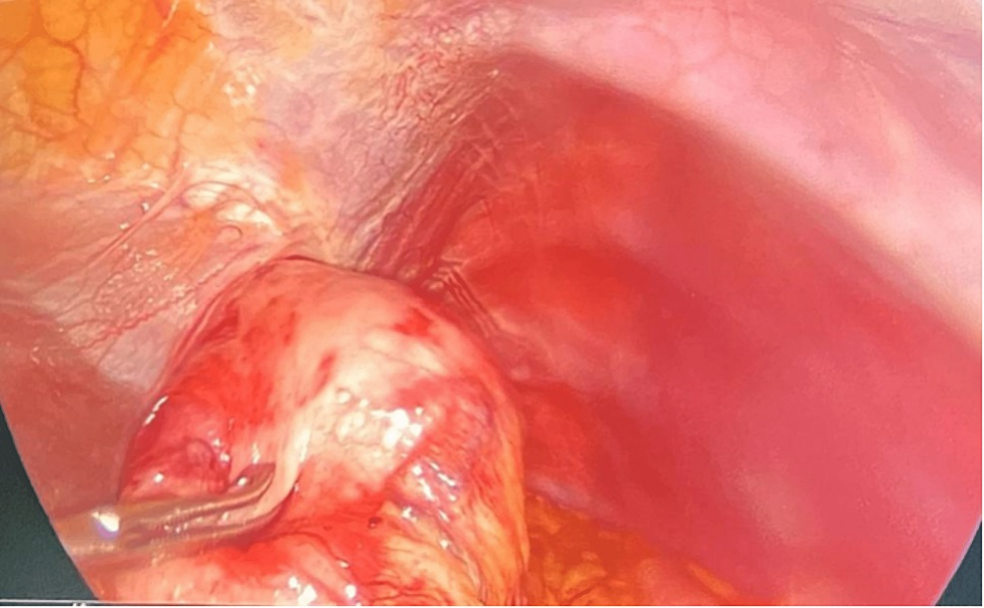
Laparoscopic view of the stomach Laparoscopic view showing a tumor mass that has developed within the wall of the stomach and has a nodular shape. The surface of the mass appears smooth and firm to the touch.

The anatomopathological study of the gastric mass biopsy revealed a spindle cell contingent with undifferentiated tumor proliferation. Surgical intervention was recommended for therapeutic purposes. The patient underwent laparoscopic wedge resection of the stomach. Exploration of the entire abdominal cavity did not reveal any metastatic lesions, peritoneal nodules, or fluid (Figure 3).

**Figure 4:**
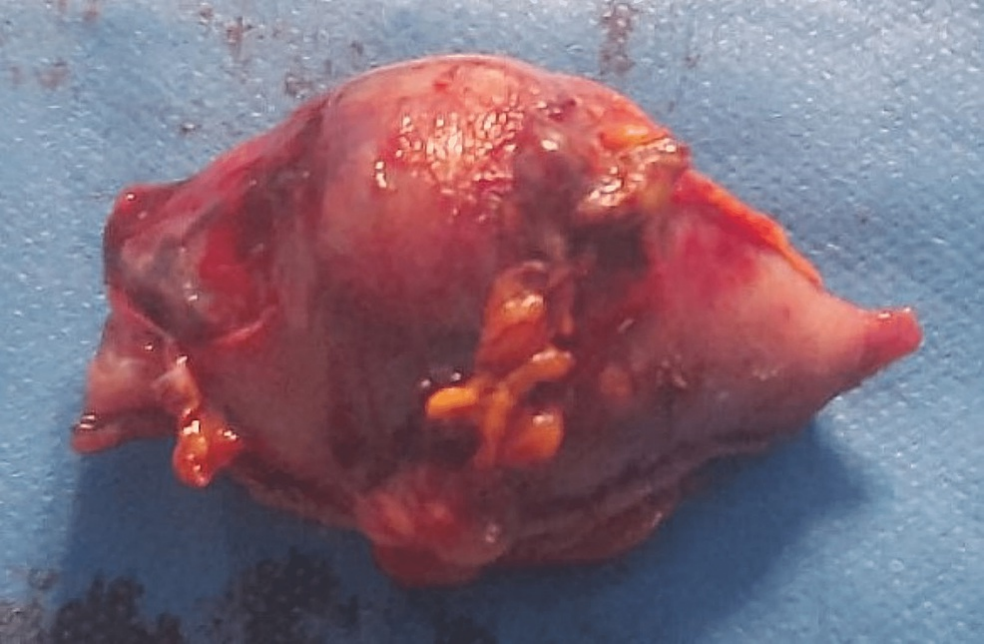
Operative specimen The tumor was a 5 cm long whitish solid mass.

A combined endo-laparoscopic approach was used to locate the tumor precisely. Mobilization of the greater curvature was achieved using a thermofusion device, and a tissue-sparing technique was used to perform a laparoscopic wedge resection with a stapling device. The surgical specimen was extracted using a bag through a 12 mm incision. No complications were observed during the post-operative period, and the patient was discharged on the fourth day after surgery (Figure 4).

**Figure 5:**
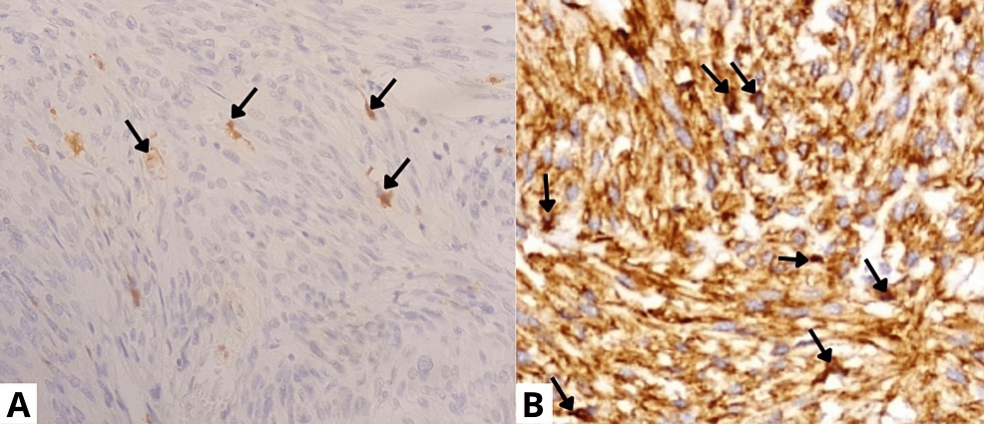
Immunohistochemistry images (A) PS100 immunostain: negative with positive internal control (arrows). (B) CD117: membranous and concentrated in a dot-like perinuclear pattern (arrows).

The pathological analysis of the surgical specimen showed a fusocellular proliferation suggestive of a 5 cm long-axis fundic GIST with a normal mucosa margin. The surgical resection was considered safe, as there were clear margins of 1 cm without any tumor cells. The tumor was classified as pT2N0M0. Immunohistochemistry analysis revealed a *CD117/KIT*+, *DOG-1*+, desmin- and PS100- phenotype (Figures 5A-5B).

Based on the consideration of the characteristics of resected localized GISTs, including a maximum diameter of 5 cm, low mitotic activity (<1/50 high-power fields), and location in the stomach, as well as complete resection and absence of perforation, the conclusion of the multidisciplinary meeting was to conduct simple surveillance by performing abdominal CT scans every 12 months. There were no complications during the postoperative period.

Case 2

**Figure 6:**
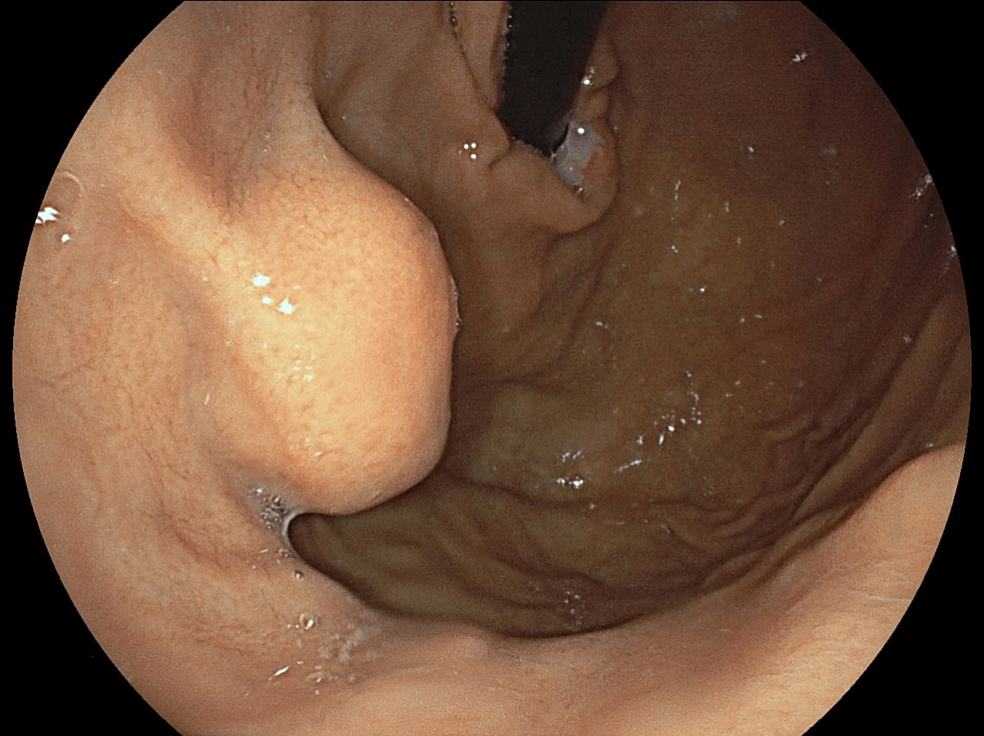
Upper gastrointestinal endoscopy Upper gastrointestinal endoscopy image showed a polypoid lesion in the fundal-subcardial region measuring approximately 2.5 cm on the long axis without ulceration.

A 65-year-old man with no significant medical history presented with complaints of profound weakness, lethargy, and lack of energy. He presented in critical condition with a blood pressure of 45/20 mmHg and a heart rate of 135 bpm. The patient was found to have severe anemia with a hemoglobin level of 4.8 g/dL. An abdominal CT scan was performed, revealing the presence of an exophytic tumor mass on the surface of the stomach, measuring 2,5 cm. An esophago-gastro-duodenal fibroscopy was performed to investigate the source of the hypovolemic shock, which revealed a polypoid fundal lesion measuring approximately 2.5 cm in the major axis (Figure 6).

**Figure 7:**
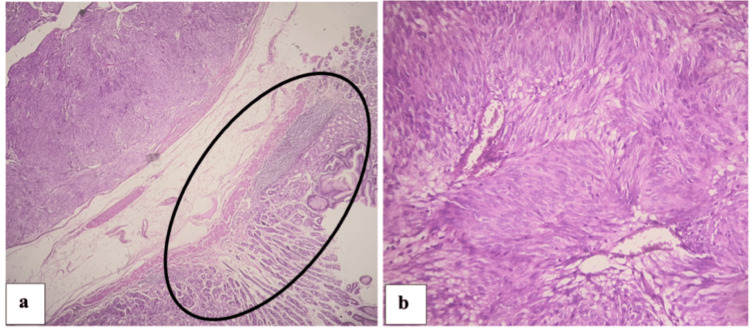
Anatomopathological study (a) GIST consisted of fascicles of spindle cells (H&E, 4X). (b) Cells showed elongated nuclei and pale eosinophilic, fibrillary cytoplasm (H&E, 20X). Note the normal fundic mucosa (circle). GIST: Gastrointestinal stromal tumor

The anatomopathological study of gastric mass biopsy was normal. As GISTs originate from the muscular layer of the digestive tract, endoscopic biopsies are frequently negative. The patient underwent a laparoscopic gastrectomy using a similar setup to the previous case. General anesthesia was induced, and a pneumoperitoneum was created using an open approach. Trocar placement was also similar to a four-trocar approach. A tissue-sparing wedge resection was performed using an Endo-GIA stapling device (Endo-GIA 45stapler; U.S. Surgical Corp. Norwalk, CT). Hemostasis was checked, and a drain was left near the cut surface. The post-operative period was uneventful, and the patient was discharged home on the third postoperative day. An anatomopathological study of the surgical specimen revealed a fusocellular tumor proliferation, with normal mucosa margin and safe surgical resection margins of 1 cm from the edge of the tumor. The final diagnosis was GIST. The tumor was classified as pT1N0M0 (Figures 7a, 7b).

**Figure 8:**
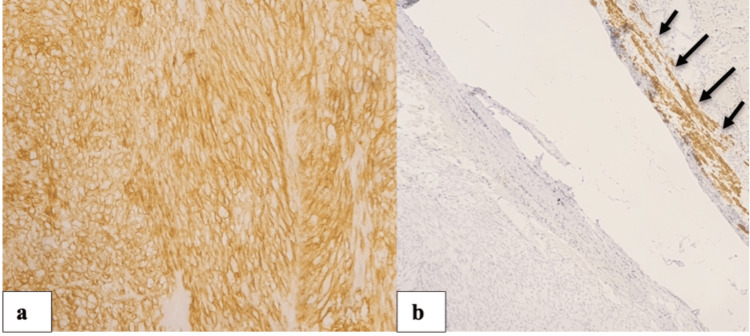
Immunohistochemistry images (a) DOG-1 immunostain, note the diffuse staining pattern. (b) GIST tumor with desmin immunostain; negative staining helps in differentiating it from leiomyoma. Note the positivity of internal control represented by normal smooth muscular fibers (arrows).

Immunohistochemistry revealed a *CD117/KIT*+, *DOG-1*+, desmin- and PS100- phenotype (Figures 8a, 8b).

The conclusion of the multidisciplinary meeting was simple surveillance by CT-abdominal every 12 months.

## Discussion

GIST is a rare mesenchymal stromal tumor that arises from Cajal cells, also known as the intestinal pacemakers, with a prevalence of 0.1-3% of all gastrointestinal tumors [6]. However, it represents the most common mesenchymal tumor of the digestive tract, accounting for 80% of cases [7]. GISTs can occur anywhere in the gastrointestinal tract but are predominantly found in the stomach or bowel [2]. The median age reported is about 60 years, with a male-to-female ratio ranging from 1.02 to 1.7 [7]. Symptoms are not specific, and patients can be asymptomatic or present with abdominal pain, weight loss, asthenia, and a feeling of a "ball in the stomach" [6]. Gastrointestinal bleeding and hypovolemia are the most dangerous complications, with some studies suggesting that tumor invasion of the mucous layer results in ulceration, while tumor rupture occurs primarily in the serosa [5,6].

The diagnosis of GIST is supported by an abdominal contrast-enhanced computed tomography (CT) scan and upper gastrointestinal exploration, while definitive diagnosis requires endoscopic ultrasonography (EUS) and pathological examination. The main differential diagnoses include leiomyoma, leiomyosarcoma, lymphoma, melanoma in the metastasis stage, and schwannoma [8]. Immunohistochemistry is used to differentiate gastric schwannomas from GISTs and leiomyomas, with the *CD117/KIT*+ (95%) and *DOG-1*+ (95%) phenotypes most commonly found. The genetic study is systematically recommended to guide the imatinib protocol, with the latest European consensus proposing that mutation of the *KIT* and *PDGFRA* genes can be used to analyze and confirm the diagnosis of GISTs, especially in *CD117* negative cases [7]. Schwannoma and GIST pose diagnostic problems. Gastrointestinal endoscopy is useful for diagnosis. Pathological examination is the only way to confirm the diagnosis, while immunohistochemistry is used to differentiate gastric schwannomas from GISTs, and leiomyomas [8]. 

Emergency surgery is often required for stromal tumors with hemorrhage. Generally, non-metastatic GISTs are treated with surgery as a first-line treatment, while metastatic GISTs are treated with chemotherapy, specifically a Bcr-Abl tyrosine kinase inhibitor: imatinib [6]. Imatinib can also be used neo-adjuvantly to reduce tumor size pre-operatively for tumors that cannot be completely resected in the first instance or as a treatment for patients that have a contraindication to surgery [9]. Complete surgical resection is the gold standard in the management of GIST, with the European Society of Medical Oncology's clinical practice guidelines recognizing all GIST as potentially malignant and recommending surgical management of all GIST without metastasis. Surgery, even if incomplete, may also be indicated palliatively for symptomatic metastatic GIST [7]. Patients who are candidates for surgical removal have a better survival rate, with a 5-year life expectancy of 48-70%, whereas the median survival of non-resectable GISTs is around 12 months, with a higher risk of recurrence [7]. Imatinib is a tyrosine kinase inhibitor that is commonly used in the treatment of GIST [9]. It is indicated for the treatment of advanced or metastatic GIST, as well as for the adjuvant treatment of high-risk GIST after surgical resection [7]. Post-Imatinib patients are monitored with CT scans to ensure that the tumor is shrinking, and if the patient responds well to chemotherapy, complete surgical resection is indicated [9].

The prognosis of patients with stromal tumors depends on various risk factors, including size, location, presence of mitotic figures, and tumor rupture. The particularity of these two cases is the sudden mode of diagnosis, as the search for the cause of hemorrhagic shock led to an emergency diagnosis [10]. An abdominal CT scan showed a mass in the gastric region, which was confirmed by upper gastrointestinal exploration. The pathological examination did not reveal any specific findings, but immunohistochemistry confirmed the typical phenotype of *CD117/KIT*+ and *DOG-1*+ [11]. Both patients underwent R0 partial gastrectomy, and a simple monitoring plan was decided upon in a multidisciplinary meeting.

## Conclusions

Gastrointestinal stromal tumors (GISTs) are rare in our context and their diagnosis largely relies on histology and immunohistochemistry. This case represents an unusual presentation of GIST, which highlights the need to sensitize emergency physicians to consider GIST as a possible cause of profound anemia and to urgently request gastroenterologists to explore the digestive tract in cases of shock with deep anemia. After stabilization, upper digestive endoscopy and colonoscopy should be performed in patients with hypovolemic shock. Radiologists should be encouraged to consider GIST as a possible diagnosis based on relatively characteristic radiologic findings. Multidisciplinary management of GISTs is crucial for improving both prognosis and quality of life.
